# Diarrhoea morbidity patterns in Central Region of Ghana

**DOI:** 10.11604/pamj.supp.2016.25.1.6261

**Published:** 2016-10-01

**Authors:** Alexander Asamoah, Donne Kofi Ameme, Samuel Oko Sackey, Kofi Mensah Nyarko, Edwin Andrew Afari

**Affiliations:** 1 Ghana Field Epidemiology and Laboratory Training Programme (GFELTP), Accra, Ghana; 2 Christian Health Association of Ghana (CHAG), Ministry of Health, Ghana; 3 Ghana Health Service (GHS), Accra, Ghana

**Keywords:** Diarrhoea, morbidity, patterns, C2, poorest districts Ghana

## Abstract

**Introduction:**

Diarrhoea diseases remain a major public health threat with nearly 1.7 billion cases annually worldwide occurring in all age groups. In Ghana diarrhoea kills about 14,000 children under five years annually. We therefore analysed data to determine the morbidity pattern of diarrhoea diseases in the Central Region of Ghana.

**Methods:**

Health facility morbidity data was reviewed from 2008-2012. Monthly data on diarrhoeal diseases were extracted from District Health Information Management System database by sex, age group and districts. Data for bloody diarrhoea were extracted from monthly surveillance report forms. Data was analysed descriptively and expressed as frequencies and proportionate morbidity rates (pmr). Aberrations were determined using C2 threshold.

**Results:**

The total cases of all morbidity from 2008 to 2012 were 7,642,431. Diarrhoea diseases formed 4% (306854/7642431) of total morbidity. Children under one year (pmr= 8.4%) and males (pmr= 4.4%) were the most affected. Bloody diarrhea formed 2.2% (6835/306854) of diarrhoea cases with 0.7 %(45/6835) laboratory confirmed. Diarrhoea cases peaked from January to March throughout the study period with highest frequency 9.3% (28511/306854) in June. The mean monthly distribution of diarrhoea cases was 25571.17±1389.91. Poorest districts had significantly lower odds of getting bloody diarrhoea than non-poorest districts OR = 0.73 (95%CI = 0.70-0.77).

**Conclusion:**

Diarrhoea characterized 4% of total morbidity presenting at health facilities in the region from 2008 to 2012. The diarrhoea morbidity rate decreased with increased age. Diarrhoea was higher among non poorest districts. The rate was highest in the month of June over the five year period. Bloody diarrhoea cases were mostly untested. We recommended that stool samples should be taken for laboratory testing for bloody diarrhoea cases.

## Introduction

Globally, diarrhoeal diseases remain a major public health threat with nearly 1.7 billion cases occurring annually [1]. Diarrhoea occurs in all age groups. However, it is more common and severe in children particularly amongst bottle fed babies and malnourished children [1]. It accounts for 1 in 9 deaths in children under five years killing 2195 children every day worldwide [2] making diarrhoea the second leading cause of death in children under five years [1, 2]. In Africa, diarrhoeal diseases cause about 16% of deaths among children under five years [3]. In Ghana it is the third leading cause of death in children under five years killing about 10,000 every year [3]. Diarrhoea can have serious impact on childhood growth and cognitive development [4]. The disease shows two main clinical presentations as non bloody diarrhoea and Bloody [5] causing death by depletion of body fluids through dehydration. The infectious agents associated with diarrhoeal disease are transmitted chiefly through the faecal-oral route [6]. An estimated 94% of the diarrhoeal disease burden is attributable to the environment, and associated with risk factors such as unsafe drinking water, poor socio economic status, lack proper sanitation and poor hygiene [7, 8]. Interventions focused at improving the point-of use as against point-of-supply of water have proven to be more effective in curbing diarrhoea diseases caused by unsafe water [9]. The patterns of diarrhoeal diseases in Ghana have been shown to peak from October to March and July to August [10]. Fewer studies have been done to determine diarrhoea burden in developing countries such as Ghana [11]. But since diarrhoea conditions are difficult to measure [12], surveillance data could be used as a basis of measurements. Moreover, records of outpatient department (OPD) diarrhoea morbidity data captured from health facilities through the districts to the region could also be analysed for some diarrhoea measurements. Data analysis with measures of incidence has been shown to give better diarrhoea measurements than prevalence measures [13]. Proportionate rate measurements [14] could therefore be used for valuable analysis of diarrhea monthly morbidity data available. In Ghana, diarrhoea is endemic particularly amongst children [3]. Surveillance data and outpatient morbidity data are captured on surveillance forms and into the District Health Information Management Systems (DHIMS) software respectively. Meanwhile these data are not analysed in detail for appropriate measurements of diarrhoeal diseases at the regional levels. Detailed analysis of the data available would help to inform policy decision for appropriate distribution of resources across the regions. But these data are mostly analysed to determine aggregates of cases to be reported to the next higher levels. Therefore little information is known about the burden, patterns and types of diarrhoea morbidity across the regions of Ghana including the Central Region. As such appropriate preparedness plan and proper distribution of resources for efficient control of diarrhoea in the region is difficult to establish. Diarrhoea data was therefore analysed to determine the pattern of diarrhoeal diseases in the Central Region of Ghana.

## Methods

### Study design

Review of health facility diarrhoea morbidity data from 2008-2012 in the DHIMS was done.

### Study site and population

The study was conducted in the Central Region which occupies about 6.6% of the total land area of Ghana. The region is 63% rural and has 20 administrative districts with Cape Coast as its capital [15]. It has a population of 2,201,863 with a sex ratio of 1:1 and a population density of about 215 inhabitants per square kilometre [16]. The region enjoys two main raining seasons in a year. The major raining season occurs in the months of April to July, peaking in June. The minor raining season peaks in October and spans the months of September to November [15].

### Data collection

Monthly Out Patient Department (OPD) morbidity data from 2008 to 2012 for all disease conditions reported in the Central Region were extracted from the District Health Information Management System (DHIMS) database. Variables collected were sex and age group from each of the 20 districts of the Central Region. Aggregates of monthly surveillance data for bloody diarrhoea were extracted from monthly surveillance report forms for the period 2008 to 2012 in the region for each district to determine its proportion of the diarrhoeal diseases.

### Data analysis

Descriptive analysis of the data was done and expressed as frequencies and proportionate morbidity rates (pmr) of diarrhoea diseases using Microsoft Excel Software. Aberrations in the diarrhoea cases recorded were determined using C2 threshold.

### Ethical issues

Permission to use the data from surveillance report forms and DHIMS was obtained from the Central regional director of health services before the start of the study. The data records were also captured without traceable identities of cases.

## Results

A total of 7,642,431 cases were recorded from 2008 to 2012. The least cases 11.2% (857685/7642431) were recorded in 2008 while the highest cases 30.3% (2315953/7642431) were recorded in 2012. Of the 7642431 cases, 58.4% (4465208/7642431) were females with cases under five years forming 21% (1607192/7642431). Of the 1607192 cases, 31% (497531/1607192) were under one year old. Records of morbidities were higher (63%) in districts of higher socio economic status (non poorest) than in districts with poor socio economic status (poorest). Diarrhoea diseases formed 4% (306854 /7642431) of all total morbidity cases seen from 2008 to 2012. Females formed 54% (165567/306854) of the diarrhoea cases. Children under five years formed 36.4% (111780/306854) with 37.2% (41614 /111780) under one year. Meanwhile, Bloody diarrhoea formed 2.2% (6835 /306854) of the diarrhoea cases. Less than one percent 0.7% (45/6835), of the bloody diarrhoea cases was laboratory confirmed over the 5 year period. The confirmed cases were all recorded in June 2012 from two districts-Asikuma Odoben Brakwa (42/45) and Awutu Senya (3/45). The proportionate morbidity rates (pmr) of diarrhoea and bloody diarrhoea were higher (4.0% and 2.5% respectively) in districts of higher socio economic status (non poorest) than in districts with poor socio economic status (poorest). Poorest districts had significantly lower odds of getting bloody diarrhoea than non poorest districts OR=0.73 (95%CI = 0.70-0.77). From 2008 to 2012, the least 9.7% (29738 /306854) diarrhoea cases were recorded in 2008 while the highest with 33.6% (103163/306854) cases was recorded in 2012. The highest 9.3% (28511/306854) cases were recorded in June whilst the least with 7.6% (23368/306854) was recorded in December. The mean of diarrhoea cases per month for the five year period was 25571.2±1399.9 forming 8.3% of diarrhoea. Table 1 shows the distribution of diarrhoea cases by sex, age groups and diarrhoea types in the Central Region from 2008 to 2012. Over the 5 year period, males showed a higher proportionate morbidity of 4.4% than females. The age distribution showed a record of the highest proportionate morbidity (8.4%) in age group less than one year old before age 1-4 years old. Bloody diarrhoea formed 2.2% of the diarrhoea cases.

**Table 1:** Characteristics of diarrhoea cases in Central Region, 2008 - 2012

Characteristics	Years					TOTAL	Total Morbidity	(pmr) %
	2008	2009	2010	2011	2012			
	n	n	n	N	n	n		
**SEX**								
Males	13959	22450	25660	32699	46519	141287	3177223	4.4
Females	15779	25004	30391	37749	56644	165567	4465208	3.7
TOTAL	29738	47454	56051	70448	103163	306854	7642431	4.0
**Age groups**								
<1	5300	7618	7155	8528	13013	41614	497531	8.4
1-4	6855	10456	12618	16188	24049	70166	1109661	6.3
5-9	2401	3817	4549	5778	8581	25126	658533	3.8
10-14	1981	2982	3394	5929	6486	20772	490654	4.2
15-17	1540	2246	2879	4328	5052	16045	413665	3.9
18-19	1249	2176	2915	3358	5014	14712	418604	3.5
20-34	3940	7482	8847	10565	16647	47481	1388616	3.4
35-49	2567	4426	5302	6310	9960	28565	998102	2.9
50-59	1397	2256	3044	3727	5629	16053	622834	2.6
60-69	976	1717	2343	2454	3925	11415	466736	2.4
≥70	1532	2278	3005	3283	4807	14905	577495	2.6
TOTAL	29738	47454	56051	70448	103163	306854	7642431	4.0
**Diarrhoea types**								
Bloody Diarrhoea	1264	1047	781	1705	2038	6835		2.2
Non-bloody Diarrhoea	28474	46407	55270	68743	101125	300019		97.8
**Total**	29738	47454	56051	70448	103163	306854		4.0

pmr - Proportionate morbidity rate

**Figure 1:**
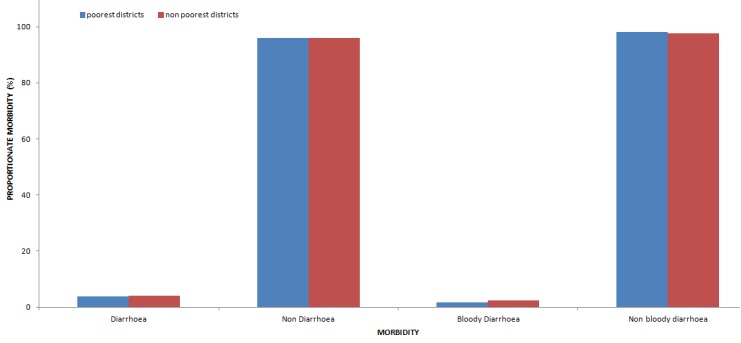
Comparison of diarrhoea OPD morbidity between poorest and nonpoorest districts in Central Region, Ghana, 2008 - 2012

Description of diarrhoea OPD morbidity by place (Figure 1) shows that, diarrhoea and bloody diarrhoea incidence was higher in non poorest districts over the 5 year period.

**Figure 2:**
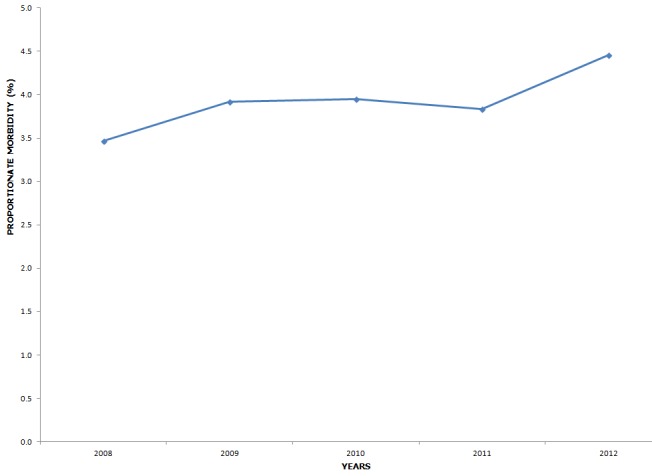
Yearly Diarrhoea OPD morbidity trends - Central Region, Ghana, 2008 - 2012

Description of diarrhoea cases by time (Figure 2) shows that, the yearly diarrhoea disease OPD morbidity increased slightly over the 5 year period with proportionate morbidity from 3.5% to 4.5%.

**Figure 3:**
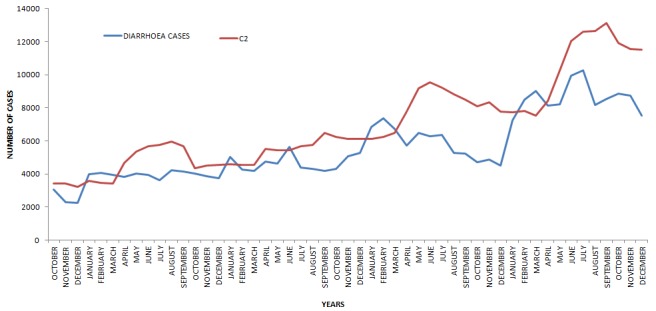
Aberrations detected from C2 threshold

Detection of aberrations with c2 threshold (Figure 3) shows that, the C2 threshold curve exposes three major aberrations in 2009, 2010 and 2012. The curve for diarrhoeal cases showed a progressively higher undulating trend with cases above 2000 per month over the 5 year period (2008-2012). The curve for the diarrhoea cases shows a pattern of increase in cases from January to March consistently from 2008 to 2012.

**Figure 4:**
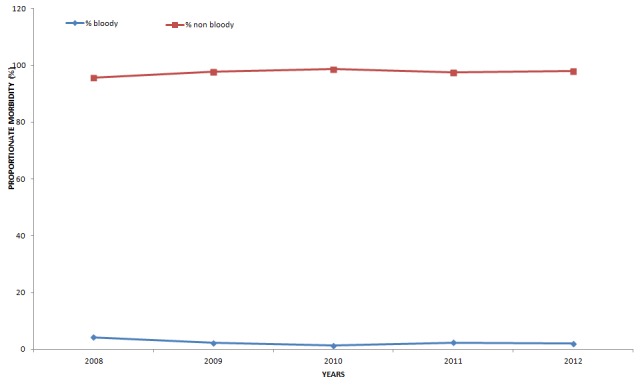
Trends of bloody and non-bloody diarrhoea types in the Central Region of Ghana, 2008 - 2012

Comparison of yearly trend of diarrhoea types (Figure 4) shows that, the curves for bloody and non bloody diarrhoea are fairly constant over the five year period. The curve of non bloody diarrhoea is far higher than that of bloody diarrhoea.

## Discussion

This study provides some evidence that four out of every one hundred cases of morbidity reported at the health facilities in the Central Region of Ghana are diarrhoeal diseases. Diarrhoeal diseases in the region have increased slightly by a proportional morbidity of one percent from 3.5% in 2008 to 4.5% in 2012 over the five year period. This finding contrasts that found by Emina et al, (2012) [17] in which the overall prevalence of diarrhoea decreased by 26 percent from 22.1% in 2001 to 16.4% in 2007 in the Democratic Republic of Congo. The morbidity rate was highest in children under one year and lowest for adults in the age group 60-69 years. Thus among children under five years and higher age groups, morbidity pattern of diarrhoea shows a decreasing rate with increasing age. This finding is consistent with that found by Ahmed et al, (2008) [18] in their prevalence study of diarrhoea among children under five in India where the diarrhoeal prevalence rates of children aged 6-11 months were higher (49.1%) than children 48-59 months (15.7%). Similarly, a study conducted by Karambu et al, (2013) [19] in Kenya showed lower rates for children over four years old than children between 12 months and 48 months old. Similar findings in children 6-11 months were found by Banerjee B, Hzra S and Bandyopadhyay D (2004) [20] and El-Gilany AH and Hammad S (2005) [21]. This pattern of decreasing morbidity with age is as a result of immature immune system of the younger children as immunity acquired from mothers decline and possible contamination of weaning foods being introduced to them occur [18]. Moreover, at such younger ages, children begin to crawl and the probability to get their fingers contaminated and put into their mouth when teething, is high [18]. However, lower rates among the higher age groups may be because they might have adapted to the environment and the immune system matured to a larger extent as described by Ahmed et al, (2008) [18]. Males were found to have a higher rate of diarrhoea morbidity than females from the study. This result is in conformity with Ahmed et al, (2008) [18] in their study which showed that, male gender (10.4%) was significantly more susceptible to diarrhoea than females (8.1%) [17]. This might be due to the finding that, females are more conscious of hygienic practices and hence maintain personal hygiene than males as reported by Mhaske Mayavati S. et al, (2013) [22] in their study in which hygiene score for skin and clothes was significantly poor in males compared to female school children in India. But this finding is in contrast with that of Syed MS. et al, (2003) [23] in which females had marginally higher prevalence of diarrhoea than males in their study of estimating the prevalence and correlates of diarrhoea among children under three years in remote rural villages of South Pakistan. The difference in these two studies [22, 23] might be due to the calibre of study subjects and the sample size used. The incidence of diarrhoea varied across the various districts of the Central Region over the five year period. The proportionate morbidity rate of diarrhoea was found to be higher in non poorest districts than the poorest districts identified by the poverty index reported in 2006 [24]. This result contrasts other studies which have found increased diarrhoea to be associated with low socio economic status. Therefore this finding of contrast between poverty and low incidence of diarrhoea is in conformity with the study conducted in the Democratic Republic of Congo by Emina JBO. and Kandala NB. (2012) [17] in which the decrease in diarrhoea prevalence was in contrast to the generalized poor living conditions in that population. The mean of diarrhoea cases per month was found to be 8.3% for the five year period. This result was in conformity with the study conducted by Njuguna J. and Muruka C. (2011) [25] in Kenya where the mean monthly prevalence was found to be 8%. The overall incidence of diarrhoea was found to be highest in the month of June (9.3%) which is the peak of the major rainy season in the Central Region (DHS, 2008). This finding however contrasts that of Armah GE. et al, (1994) [10] in their study conducted in Ghana which showed that diarrhoea peaks in two seasons, October to March and July to August. This difference might be due to climate change. But, the finding also contrasted the studies conducted in India by Ahmed et al,(2008) [18], Banerjee, B., Hzra S. and Bandyopadhyay D. (2004) [20] and in Egypt by El-Gilany AH. and Hammad S. (2005) [21] where prevalence of diarrhoea was higher during the summer months. The difference in this finding may be due to the different study settings and design. Meanwhile, a pattern of increases in diarrhoea cases occurred from January to March throughout the five year period. This might be due to batch reporting practiced by some data managers due to the festivities and holidays that come before that period. In this practice, data are mostly compiled and reported late after the holidays together with current data leading to false increases in cases. Moreover, the data for the five year period was used to develop a C2 threshold to detect aberrations that may occur during surveillance. This threshold exposed aberrations in 2009, 2010 and 2011 which were missed and therefore not investigated. Bloody diarrhoea was found to form 2.2% of diarrhoea cases in the region. However, diarrhoea cases of bloody diarrhoea were mostly managed without laboratory testing of stool samples to isolate the causative pathogen. This finding therefore showed that, surveillance activities of bloody diarrhoea was incomplete among the districts in the Central Region over the five year study period. The limitation of this study was that, only OPD diarrhoea morbidity data was used as captured from the DHIMS. As such admitted diarrhoea cases were not considered during the analysis and therefore the findings might not reflect total diarrhoea cases in the region. However, we recommend that, the deputy director of public health should educate and encourage prescribers to take stool samples for laboratory testing for all suspected cases of bloody diarrhoea as required by the surveillance system.

## Conclusion

Diarrhoea characterized 4% of total morbidity presenting at health facilities in the region from 2008 to 2012. Males and children less than one year old are the most commonly affected. The morbidity rate was found to decrease with increased age and varied across the 20 districts of the region. The rate was highest in the month of June for the five years. Diarrhoea with blood accounted for 2% of diarrhoea morbidity and was significantly higher in non poorest districts than poorest districts.
